# Academic and social-behavioral assessment in a prospective cohort of normocephalic school-aged children with antenatal Zika virus exposure

**DOI:** 10.1016/j.ijid.2025.108026

**Published:** 2025-08-20

**Authors:** Karen Kikuta, Christopher Justin Hernandez, Andrea Dunshee de Abranches, Luana Damasceno, José Augusto de Britto, Claudia Jardim Duarte, Zilton Vasconcelos, Andrea Zin, Patricia Brasil, Maria Elisabeth Moreira, Karin Nielsen-Saines

**Affiliations:** 1 David Geffen School of Medicine at UCLA, Los Angeles, CA, USA; 2 Fundação Oswaldo Cruz, Rio de Janeiro, RJ, Brazil

**Keywords:** Congenital infections, Academic outcomes, Zika virus, School performance, Strengths and difficulties questionnaire

## Abstract

**Objectives:**

Long-term outcomes in children with antenatal Zika virus (ZIKV) exposure without microcephaly are unknown. We assessed academic and social-behavioral outcomes among normocephalic school-aged children with antenatal ZIKV exposure and controls.

**Methods:**

School-aged children exposed to Zika (ZEC) and unexposed controls (ZUCs) were recruited in Rio de Janeiro, Brazil. The academic performance and strengths and difficulties questionnaire was evaluated through parental interviews. Clinical/demographic data were abstracted from medical records/interviews. Potential associations with trimester of infection, early neurodevelopment, and neuro-sensory findings were explored in ZEC.

**Results:**

In total, 147 children were enrolled: 78 ZEC and 69 ZUCs. The mean age for ZEC was 8.1 years and the mean age for ZUCs was 7.7 years; 50.0% of ZEC and 44.9% of ZUCs were male. Reading (21.8% vs 7.2%, *P* = 0.0193) difficulties, writing (20.5% vs 5.8%, *P* = 0.0144) difficulties, and suspected/diagnosed neurodevelopmental disorders (39.7% vs 17.4%, *P* = 0.0036) were greater in ZEC than ZUCs. ZEC had higher scores in total difficulties (13.32 vs 10.10, *P* = 0.0099), emotional symptoms (4.33 vs 2.90, *P* = 0.0011), and hyperactivity (4.95 vs 3.41, *P* = 0.0037). ZIKV exposure was a predictor of reading (adjusted odds ratio [aOR] = 3.39, 95% confidence interval [CI] = 1.17–9.79), writing (aOR = 4.02, 95% CI = 1.27–12.74), emotional (aOR = 3.51, 95% CI = 1.72–7.18), and hyperactivity (aOR = 2.43, 95% CI = 1.18–4.98) problems. Early developmental delay was more frequent in ZEC with academic difficulties (*P* = 0.0018).

**Conclusions:**

Antenatal ZIKV exposure is associated with increased risk of academic difficulties, neurodevelopmental diagnoses, and social-behavioral problems in school-aged normocephalic children.

## Introduction

Zika virus (ZIKV) is a mosquito-borne virus that was found to be a significant human pathogen in 2007 after outbreaks in multiple continents, including the Americas, Asia, and Africa [1, 2]. Between May 2015 and December 2016, over 700,000 cases were reported in the Americas [3, 4]. Brazil was particularly impacted, with over 200,000 ZIKV cases reported by the end of 2016, along with a significant 20-fold increase in microcephaly cases reported by health surveillance systems [4–7]. Given its significant repercussions, understanding the full spectrum of the vertical transmission of ZIKV became a crucial task. *In utero* ZIKV infection has since been associated with multiple negative outcomes, including fetal loss, intrauterine growth restrictions, and neurological abnormalities [8]. Its most severe manifestation is defined as congenital ZIKV syndrome (CZS), with an emphasis on features of severe neurological, structural malformations to the brain and neuro-sensory abnormalities, leading to hearing and visual deficits [9, 10].

Although substantial evidence has been gathered on microcephalic or otherwise symptomatic infants, there continues to be a lack of evidence on long-term outcomes in normocephalic asymptomatic children with confirmed antenatal ZIKV exposure. More recently, a high frequency of anatomical and neurodevelopmental abnormalities has been identified in children without microcephaly up to 4 years of age [4, 11–15]. Additional studies extend our understanding into the school-age period, showing that children without CZS exposed to ZIKV *in utero* may experience lower executive control, mood, adaptive functioning, and full-scale intelligence quotient scores than children without exposure [16, 17].

Considering these recent findings and the limitations of the existing literature, our study aimed to assess a larger sample of school-aged normocephalic children with laboratory-confirmed antenatal ZIKV exposure compared with unexposed peers in Rio de Janeiro, Brazil. We assessed long-term academic, social, behavioral, and emotional outcomes while also examining potential risk factors, such as maternal health during pregnancy and early childhood development, which could influence these trajectories.

## Methods

### Study population and site

This study followed up a prospective cohort of children exposed to *in utero* ZIKV during the 2015–2016 epidemic in Rio de Janeiro, Brazil. Study participants had laboratory-confirmed ZIKV exposure, which was performed in the birthing parent and/or infant through reverse transcription–polymerase chain reaction of maternal blood or urine samples and/or amniotic fluid during pregnancy and in neonatal urine, serum, and/or cerebrospinal fluid samples. We excluded symptomatic children with microcephaly and other features of CZS, such as intracranial calcifications and other congenital infections. The ZIKV study group was followed up longitudinally at the Instituto Fernandes Figueira (IFF) of the Oswaldo Cruz Foundation (FIOCRUZ), which is a major referral center for ZIKV cases in Rio de Janeiro.

Age-matched controls were included in this study. Control children were recruited either from the same site (IFF/FIOCRUZ) as ZIKV pediatric cases with antenatal ZIKV exposure or were composed of healthy children raised in the same family and/or environment as the ZIKV study group children (either a close sibling or cousin). At IFF/FIOCRUZ, the unexposed ZIKV control group had no antenatal exposure to ZIKV. These children were followed up longitudinally at the clinical site after their initial recruitment for the National Institutes of Health–funded International Prospective Observational Cohort Study of Zika in Infants and Pregnancy (ZIP study) [18]. In that study, pregnant women underwent rigorous prospective monitoring with twice-monthly polymerase chain reaction testing throughout pregnancy and consistently remained negative for ZIKV infection. For the family/environmental controls, ZIKV exposure was excluded by birth date, which occurred outside the epidemic period, and a negative maternal history of ZIKV during pregnancy.

### Data collection

We crafted a questionnaire that included an assessment of academic performance, as well as the Strengths and Difficulties Questionnaire (SDQ). The academic performance assessment included items about school type (public vs private); grade level; writing, reading, and mathematical abilities; school attendance; and neurodevelopmental difficulties (Supplementary Material 1). Sensory screening (history of vision and hearing evaluations) was also included in this part of the questionnaire because impairment in these areas can affect academic skills and survey responses (Supplementary Material 1). Children with untreated and/or severe sensory disturbances were excluded. Social, behavioral, and emotional well-being were assessed through the SDQ (single-sided version without impact supplement for parents or teachers of children aged 4–17 years) [19]. We used the Brazilian Portuguese version, which has been translated and validated in Brazilian populations [20]. SDQ results can be categorized as normal, borderline, or abnormal based on established normative bandings, which were originally derived from a UK population-based survey, designed so that approximately 80% of children score in the normal range, 10% borderline, and 10% abnormal [21]. The questionnaire was administered through phone or in-person interviews with the children’s parent or guardian, between February and April of 2025.

Additional data on early neurodevelopment (Bayley Scales of Infant and Toddler Development III) and neuro-sensory problems (ophthalmologic exam, brainstem evoked response audiometry, and audiometry) of children exposed to ZIKV that had been followed up longitudinally at IFF/FIOCRUZ were abstracted from medical records. Demographic information for all children was abstracted from medical records and interviews.

### Questionnaire interpretation

For the academic assessment, reading, writing, and math difficulties were recorded as positive (“yes”) if the child was unable to develop expected skills for the grade level based on school evaluations and feedback, comparison with peers in the class, and need for additional assistance. Attendance was assessed as higher or lower than 80% of the time in the previous year. Neurodevelopmental difficulties included the presence of diagnoses that might affect academic performance and daily life, including attention-deficit/hyperactivity disorder, autism spectrum disorder, learning disorders (e.g. dyslexia), intellectual disability, and significant trauma or anxiety symptoms. Children had none, suspected, or diagnosed difficulties—the last two determined by explicit suspicion and/or ongoing evaluation by a health care professional at a clinic or the school attended by the child, without or with a final diagnosis, respectively.

The SDQ consists of 25 items divided among five subscales: emotional symptoms, conduct problems, hyperactivity, peer relationship problems, and prosocial behavior. Each item is rated on a three-point scale: 0 (not true), 1 (somewhat true), or 2 (certainly true). For scoring, each subscale ranges from 0 to 10. In addition, the first four subscales are summed to generate a total difficulties score, ranging from 0 to 40. Higher scores indicate greater difficulties for the first four subscales and the total difficulties. The prosocial behavior subscale is scored separately, with higher scores reflecting stronger social skills. Interpretation involves comparing each final score with established norms to determine whether they fall within normal, borderline, or abnormal ranges.

### Early neurodevelopmental and neuro-sensory screening

Neurodevelopment was assessed in our ZIKV-exposed cohort using the Bayley Scales of Infant and Toddler Development, 3rd Edition (Bayley-III) between birth and 37 weeks of age. Cognitive, language, and motor scores were obtained and averaged. Standardized mean scores lower than 85 (1 SD below average) and 70 (2 SD below average) were categorized as “at risk” and “developmental delay,” respectively. Ophthalmologic exam data were obtained in our ZIKV cohort between birth and 19 months of age. The exam included assessment of the external eye and fundoscopy. Positive findings in either of these components were categorized as an abnormal ophthalmologic exam. Brainstem evoked response audiometry (BERA) data were obtained in our ZIKV cohort between 1 and 42 months of age, whereas school-age audiometry was obtained between 7 and 9 years of age. Abnormal BERA results included no wave V ≤40 dB hearing level, prolonged/absent waves, or latency delays; abnormal school-age audiometry results included misses in any frequency ≥20 dB hearing level in one or both ears. All screenings were completed by trained specialists at IFF/FIOCRUZ, including neuropsychologists, ophthalmologists, and phono audiologists.

### Statistical analysis

Data were de-identified by case number assignment. Differences in results between cases and controls were assessed through Fisher’s exact test and Student’s unpaired *t* -test. Logistic regression analysis was performed to evaluate predictors of difficulties that were significant in academic performance and social-behavioral outcomes. Early neurodevelopmental and neuro-sensory data within ZIKV-exposed group were analyzed using the chi-square test. We used the Holm–Bonferroni method to adjust for multiple comparisons across five correlated outcomes because it controls the family-wise error rate while maintaining more power than the standard Bonferroni correction. We selected this approach to ensure strong type I error control for these confirmatory analyses. Software used include GraphPad Prism 10 and R-Studio.

## Results

A total of 147 children were included in the study, composed of 78 with confirmed antenatal ZIKV exposure and 69 in the control group (Supplementary Figure 1). Among the controls, 46 were from the same living environment, and 23 were recruited from the same clinical site (Supplementary Figure 1).

As seen in Supplementary Table 1, the mean age of all participants was 7.96–8.1 years in the ZIKV group (range: 7–9) and 7.7 years in controls (range: 5–13). The sex distribution was similar across groups (50.0% males in the ZIKV group vs 44.9% males among controls) (Supplementary Table 1). Regarding birth history, most children were born at term, with a slightly higher percentage of preterm births in the ZIKV group than in controls (18.7% vs 13.0%) (Supplementary Table 1). Delivery by cesarean delivery was also more common in the ZIKV group than in controls (72.0% vs 56.5%) (Supplementary Table 1). The frequency of maternal age older than 35 years at birth was similar across groups (28.2% in ZIKV vs 28.4% in control children) (Supplementary Table 1). Maternal comorbidities, including hypertension, diabetes, and chorioamnionitis, were higher in controls than in the ZIKV group (26.1% vs 20.5%) (Supplementary Table 1).

Within the ZIKV group, the timing of maternal infection was highest in the second trimester of pregnancy (48.7%), followed by first (28.2%) and third (18.0%) trimesters (Supplementary Table 1). In 5.1% of the cases, the timing of infection was unknown because the mother was asymptomatic (Supplementary Table 1).

The academic assessments revealed that reading and writing difficulties were significantly more prevalent in the ZIKV group of children (Table 1). Reading difficulties were reported in 21.8% of children exposed to ZIKV compared with 7.2% of controls (*P* = 0.0193), with children exposed to ZIKV having a 3.6 times higher risk of reading difficulties (Table 1). Writing difficulties were seen in 20.5% of children exposed to ZIKV vs 5.8% of controls (*P* = 0.0144), with children exposed to ZIKV having a 4.2-fold higher risk of exhibiting writing difficulties (Table 1). Diagnosed or suspected neurodevelopmental difficulties were identified more often in the ZIKV group than in controls (39.7% vs 17.4%, *P* = 0.0036). The absence of ZIKV exposure was a protective factor against neurodevelopmental difficulties (odds ratio [OR]: 0.32) (Table 1). School type, attendance, and math difficulties were similar between both groups (Table 1).

**Figure 1.**
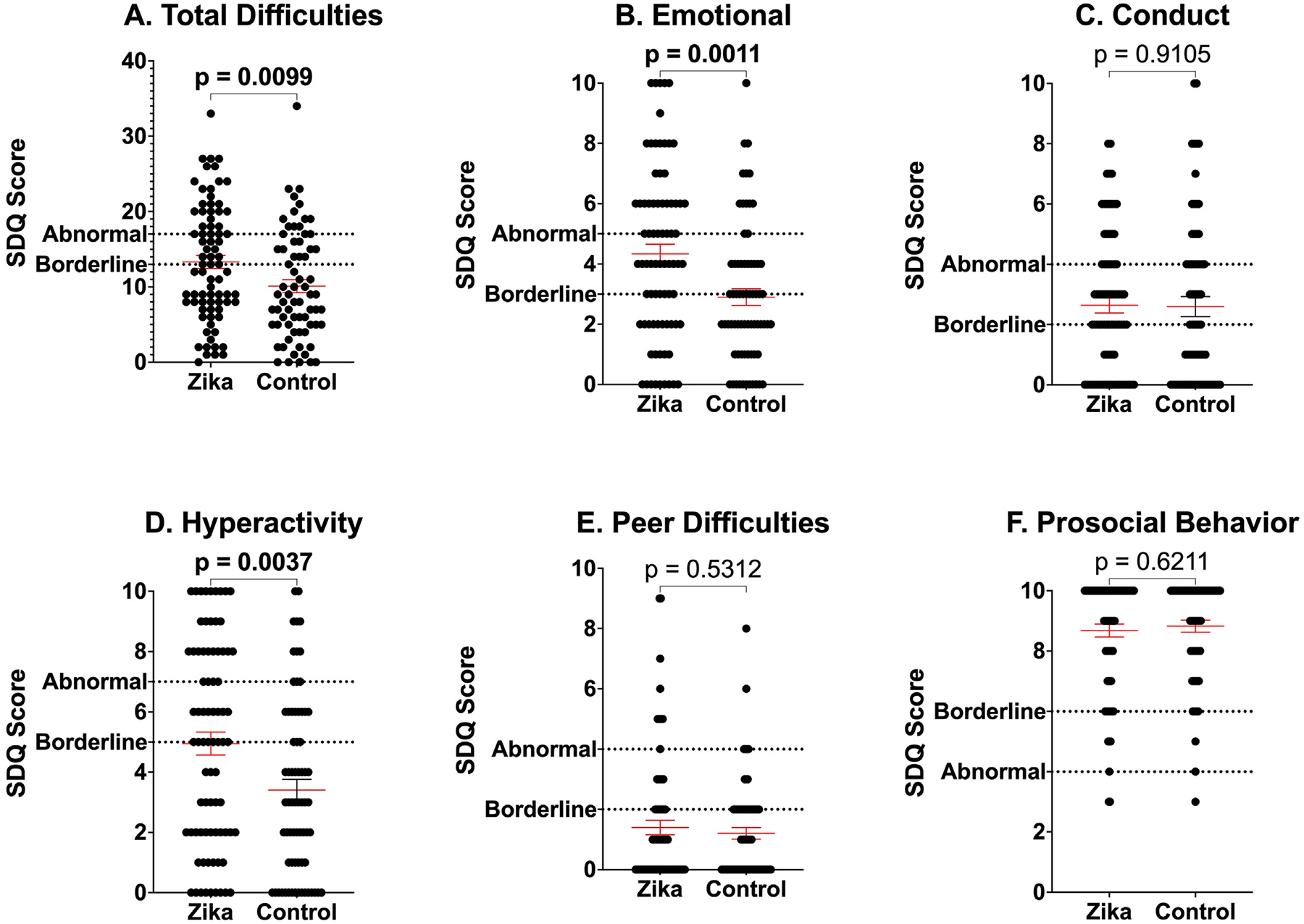
SDQ scores. Mean SDQ subscale and total difficulty scores for Zika virus group and control group. Data shown as mean ± SEM. Dotted lines indicate borderline and abnormal thresholds. SDQ, strengths and difficulties questionnaire.

On the SDQ, the children exposed to ZIKV showed significantly higher mean scores in total difficulties (13.32 vs 10.10, *P* = 0.0099), emotional symptoms (4.33 vs 2.90, *P* = 0.0011), and hyperactivity (4.95 vs 3.41, *P* = 0.0037) than unexposed controls (Figure 1, Supplementary Table 2). No significant differences were observed between groups in conduct problems, peer difficulties, or prosocial behavior scores (Figure 1, Supplementary Table 2).

In the logistic regression analyses, ZIKV exposure was a significant predictor of reading and writing difficulties, with over three times greater odds of reading difficulties and a four-fold higher odds of writing difficulties in the ZIKV group of children than in controls (adjusted OR: 3.39, 95% confidence interval [CI]: 1.17–9.79 and adjusted OR: 4.02, 95% CI: 1.27–12.74, respectively), (Table 2). Maternal age, child’s age, child’s sex, prematurity, delivery type, maternal comorbidities, and private schooling were not significantly associated with academic outcomes (Table 2).

Regarding social-behavioral outcomes that showed significance between groups, ZIKV antenatal exposure was also a significant predictor of emotional and hyperactivity problems (adjusted OR: 3.51, 95% CI: 1.72–7.18 and adjusted OR: 2.43, 95% CI: 1.18–4.98, respectively) (Table 3). Prematurity emerged as a predictor of emotional symptoms (OR: 2.85, 95% CI: 1.09–7.43); however, this association was not significant in the adjusted models (Table 3). Maternal age, child’s age, trimester of ZIKV exposure, child’s sex, delivery type, maternal comorbidities, and private schooling were not significantly associated with social-behavioral outcomes (Table 3).

Children exposed to ZIKV were followed up since birth, which allowed us to review additional data on early neurodevelopmental and neuro-sensory outcomes. Among 78 children exposed to ZIKV, 62 (79.5%) had Bayley-III completed in early childhood (Table 4). Children with any academic difficulty (including reading, writing or math) were more likely to have lower Bayley-III scores early in life, indicating developmental delay (33.3% vs 2.1%, *P* = 0.0018) (Table 4). Children with any abnormal SDQ score also had higher rates of abnormal Bayley-III scores, indicating developmental delay, compared with children with normal SDQ scores (11.5% vs 0%, *P* = 0.22), although these findings did not achieve statistical significance (Table 4).

Among 78 exposed children, 67 (85.9%) had ophthalmologic exams completed in early childhood (Table 4). Children with any academic difficulty and any abnormal SDQ score had higher rates of abnormal ophthalmologic exams than their peers with normal academic and SDQ outcomes (62.5% vs 47.1%, *P* = 0.28 and 54.39% vs 30.0%, *P* = 0.15, respectively); however, these findings did not achieve statistical significance (Table 4).

Lastly, we reviewed data from auditory exams. Among 78 children exposed to ZIKV, 64 (82.1%) completed BERA, and 62 (79.5%) completed school-age audiometry (Table 4). Abnormal findings were less frequent overall, and they were not clearly associated with academic or SDQ outcomes, as seen in Table 4.

## Discussion

ZIKV is an arthropod-borne virus in the *Flaviviridae* family that proved to be a significant pathogen to infants after antenatal exposure, leading to the identification of CZS in children born during the 2015–2016 ZIKV epidemic [22]. The major signs and symptoms of CZS include microcephaly; parenchymal calcifications; ventriculomegaly; central nervous system atrophy or hypoplasia; arthrogryposis; low birthweight; and ophthalmic abnormalities such as focal pigmentary retinal mottling, chorioretinal atrophy, and abnormal visual function [13, 23, 24]. Although microcephalic and symptomatic children have been more extensively studied, data on the long-term outcomes involving normocephalic and initially asymptomatic children are lacking.

In the present study, we assessed academic and social-behavioral outcomes in school-aged children with a history of *in utero* ZIKV exposure compared with unexposed peers. Antenatal ZIKV exposure was significantly associated with higher odds of reading and writing difficulties, even after adjustment for confounders. Although math difficulties were not statistically significant, the elevated OR noted (OR: 3.61) suggests a meaningful trend that may have been limited by measurement sensitivity or sample size. In addition, we found a higher frequency of suspected or diagnosed neurodevelopmental conditions, such as autism spectrum disorder, attention-deficit/hyperactivity disorder, and learning disorders, in children exposed to antenatal ZIKV than in their unexposed peers. These findings support the recent literature. Mulkey *et al*. [16], for example, found significantly lower language and cognitive scores in preschool-aged children, which suggested persistent learning risks in this population when evaluated at an older age, as in our study. Other studies also note subtle but impactful neurocognitive and neurodevelopmental deficits in mainly younger children with a history of *in utero* ZIKV exposure, even without microcephaly [4, 11–15, 17].

The results from SDQ suggested potential challenges with self-regulation and emotional well-being. Children exposed to ZIKV showed significantly higher scores in total difficulties, emotional symptoms, and hyperactivity domains. Although SDQ is not used as a diagnostic tool, it has clinical implications with borderline and abnormal scores, prompting additional support systems—e.g. social work and therapy—and referral to pediatric mental health specialists, respectively. These findings align with recent literature that report higher rates of internalizing and externalizing behavioral problems in toddlers who were exposed to ZIKV *in utero*, delays in expressive language and socioemotional engagement, and increased behavioral dysregulation and attention deficits in school-age follow-up [10, 12, 17].

Our analysis of early neurodevelopment and neuro-sensory findings in the ZIKV-exposed cohort showed a significant association between academic difficulties and developmental delay identified by Bayley-III assessments in the first years of life, which is an expected finding. A trend toward an association between below-average neurodevelopment and social-behavioral difficulties was noted, with statistical significance likely not achieved due to the sample size. Similarly, a trend toward the presence of eye abnormalities being more frequently identified in children with academic or social-behavioral difficulties was noted. There is evidence that neurological outcomes in children exposed to antenatal ZIKV are predictive of poor early neurodevelopmental scores [4]. Proper neurological and visual functioning are critical for learning and neurodevelopment. Although audiometry data showed no strong associations, screening and longitudinal follow-up is recommended.

Increased academic and social-behavioral difficulties are potentially associated with the pathophysiology of ZIKV congenital infection. ZIKV demonstrates strong neurotropism for neural progenitor cells, especially during the first and second trimesters of pregnancy (when 76.9%, i.e. the majority of our ZIKV cohort, had maternal viral exposure) [8, 25–30]. Neurogenesis is critical for adequate development of early executive function, memory, and attention, which influence social integration, overall well-being, and classroom functioning. These processes, therefore, become vulnerable to disruption when the fetal brain is exposed to ZIKV.

The study has some inherent limitations. First, there is some heterogeneity within the control group. Although unexposed children were recruited from the same clinical site when possible, limited access to control children actively followed up at IFF/FIOCRUZ necessitated additional recruitment from the same living environment as the ZIKV-exposed group. Although this approach may have introduced variability in baseline exposures, it allowed us to maintain demographic and socioeconomic comparability. Second, we were unable to obtain complete data on additional socioeconomic factors, such as parental education and household income, which could potentially confound outcomes. Nevertheless, recruitment of control children from the same living environment or followed up at the same public institution as cases was an approach designed to mitigate confounding socioeconomic variables. Third, to the best of our knowledge, although this is the largest study to date on normocephalic school-aged children with antenatal ZIKV exposure, the sample size may have limited power to detect certain associations, such as math difficulties and subgroup analyses (e.g. maternal trimester of infection). Fourth, there was a small age range variation between exposed and unexposed children, although the mean ages were similar. This was done intentionally to ensure controls were born outside the time frame for the 2015–2016 epidemic and minimize the risk of undetected exposure. It is important to note this may influence academic expectations and neurodevelopmental maturity, although, under this scenario, this would lean toward the opposite direction because children exposed to ZIKV tended to be slightly older. Finally, the fact that the questionnaires were parent- or guardian-reported may subject answers to ascertainment bias because children exposed to ZIKV might receive more evaluations and parental attention. However, many control children were also longitudinally followed up at the same clinical site or came from similar familial and living environments, helping to mitigate this concern. In addition, neurodevelopmental disorders were only reported if they had been identified by a health care or educational professional and not based on parental suspicion alone.

Our study supports guidelines that reinforce standardized and multidisciplinary follow-up protocols. The American Academy of Pediatrics recommends the use of structured tools, such as SDQ and the modified checklist for autism in toddlers and ages & stages questionnaire, in combination with clinical history, to guide appropriate referrals and early intervention [31]. Furthermore, this study contributes to the growing body of evidence that ZIKV exposure *in utero* can have significant long-term outcomes in normocephalic and initially asymptomatic children. We found increased academic and social-behavioral challenges that support a lifespan-based model of developmental monitoring for all exposed children instead of a crisis-oriented model limited to children with overt congenital anomalies. Future public health strategies should prioritize early detection, educational support, and longitudinal multidisciplinary coordination of neurodevelopmental care for all children exposed to antenatal ZIKV.

## Supplementary Material

Supplemental Figure 1

Supplemental Material 1

Supplemental Table 1

Supplemental Table 2

Supplementary material associated with this article can be found, in the online version, at doi:10.1016/j.ijid.2025.108026.

## Figures and Tables

**Table 1:** Academic assessment: comparison of academic performance and neuro-developmental difficulties between children exposed to ZIKV and controls.

	ZIKV group n (%)	Control group n (%)	*P*-value	Odds ratio (95% confidence interval)
Number of children	78	69	-	-
School type				
Public	29 (37.2)	24 (34.8)	0.8636	1.11 (0.58 to 2.16)
Private	49 (62.8)	45 (65.2)		
Reading difficulties				
Yes	17 (21.8)	5 (7.2)	**0.0193**	**3.56 (1.33 to 9.20)**
Writing difficulties				
Yes	16 (20.5)	4 (5.8)	**0.0144**	**4.19 (1.39 to 11.97)**
Math difficulties				
Yes	11 (14.1)	3 (4.3)	0.0521	3.61 (1.02 to 12.46)
Attendance				
>80%	76 (97.4)	66 (95.6)	0.6658	1.73 (0.34 to 9.93)
Neurodevelopmental difficulties identified				
None	47 (60.3)	57 (82.6)	**0.0036**	**0.32 (0.15 to 0.70)**
Suspected	15 (19.2)	6 (8.7)		
Diagnosed	16 (20.5)	6 (8.7)		

**Table 2:** Predictors of academic difficulties.

Predictors	Reading^a,b^ OR (95% CI)	Reading Adjusted^a,c^ OR (95% CI)	Writing^a,b^ OR (95% CI)	Writing Adjusted^a,c^ OR (95% CI)
Maternal age	0.99 (0.93–1.06)		0.99 (0.93–1.07)	
Child’s age	0.96 (0.67–1.37)		0.826 (0.57–1.21)	
ZIKV exposure	**3.46 (1.19–9.95)**	**3.39 (1.17–9.79)**	**4.06 (1.28–12.84)**	**4.02 (1.27–12.74)**
Child’s sex				
Female	Reference	Reference	Reference	Reference
Male	1.39 (0.56–3.46)	1.36 (0.53–3.47)	1.12 (0.44–2.88)	1.10 (0.41–2.91)
Prematurity				
No	Reference		Reference	
Yes	1.67 (0.55–5.09)		1.36 (0.41–4.50)	
Delivery type				
CS	Reference		Reference	
Vaginal	1.33 (0.53–3.38)		1.62 (0.62–4.21)	
Maternal hypertension				
No	Reference		Reference	
Yes	0.43 (0.09–1.99)		0.83 (0.22–3.06)	
Maternal diabetes				
No	Reference		Reference	
Yes	0.79 (0.09–6.75)		0.88 (0.10–7.62)	
Private schooling				
No	Reference	Reference	Reference	Reference
Yes	1.23 (0.51–3.26)	1.30 (0.50–3.37)	1.56 (0.60–4.06)	1.55 (0.58–4.13)

CI, confidence interval; OR, odds ratio; ZIKV, Zika virus.

a Academic outcomes that had significant differences between the ZIKV group and control group were included.

b Unadjusted logistic regression is the associated impact of the predictor on dependent variable.

c Adjusted models include whichever variables were significant at the bivariate level at *P* ≤0.10.

**Table 3:** Predictors of social-behavioral difficulties.

Predictors	Total difficulties^a,b^ OR (95% CI)	Total difficulties Adjusted^a,c^ OR (95% CI)	Emotional^a,b^ OR (95% CI)	Emotional Adjusted^a,c^ OR (95% CI)	Hyperactive^a,b^ OR (95% CI)	Hyperactive Adjusted^a,c^ OR (95% CI)
Maternal age	0.95 (0.90–0.99)	0.97 (0.94–1.01)	0.97 (0.93–1.02)		0.94 (0.90–0.99)	0.97 (0.94–1.01)
Child’s age	0.92 (0.71–1.19)		1.15 (0.89–1.48)		0.85 (0.65–1.11)	
Zika exposure	1.85 (0.94–3.63)	1.95 (0.98–3.87)	**3.10 (1.57–6.14)**	**3.51 (1.72–7.18)**	**2.33 (1.16–4.70)**	**2.43 (1.18–4.98)**
Trimester						
First	Reference		Reference		Reference	
Second	0.42 (0.14–1.23)		0.47 (0.16–1.40)		0.34 (0.11–1.02)	
Third	0.57 (0.15–2.23)		2.8 (0.49–16.04)		0.26 (0.06–1.07)	
Child’s sex						
Female	Reference		Reference		Reference	Reference
Male	1.57 (0.81–3.06)		1.14 (0.59–1.19)		1.88 (0.95–3.72)	1.91 (0.95–3.86)
Prematurity						
No	Reference		**Reference**	Reference	Reference	
Yes	1.36 (0.55–3.32)		**2.85 (1.09–7.43)**	2.63 (0.95–7.24)	0.85 (0.33–2.15)	
Delivery type						
Cesarean	Reference		Reference		Reference	
Vaginal	1.33 (0.67–2.66)		0.75 (0.38–1.50)		1.29 (0.64–2.62)	
Maternal hypertension						
No	Reference		Reference	Reference	Reference	
Yes	0.79 (0.32–1.92)		2.25 (0.92–5.49)	2.23 (0.85–5.81)	0.47 (0.18–1.27)	
Maternal diabetes						
No	Reference		Reference		Reference	
Yes	0.87 (1.99–3.78)		0.65 (0.15–2.81)		0.54 (0.11–2.80)	
Private schooling						
No	Reference		Reference		Reference	
Yes	0.98 (0.49–1.96))		0.91 (0.46–1.81)		1.23 (0.61–2.48)	

CI, confidence interval; OR, odds ratio; ZIKV, Zika virus.

a Strengths and difficulties questionnaire scores with significant differences between children exposed to ZIKV and controls were included.

b Unadjusted logistic regression is the associated impact of the predictor on dependent variable.

c Adjusted models include whichever variables were significant at the bivariate level at *P* ≤0.10.

**Table 4:** Association between early neurodevelopmental and neuro-sensory findings and academic and SDQ outcomes^a^.

	Any academic difficulty n (%)	No academic difficulties n (%)	*P*-value	Borderline or abnormal SDQ score n (%)	Normal SDQ scores n (%)	*P*-value
Bayley	n = 15	n = 47		n = 52	n = 10	
Normal	5 (33.3)	24 (51.1)	**0.0018**	22 (42.3)	7 (70.0)	0.2196
At Risk	5 (33.3)	22 (46.8)		24 (46.2)	3 (30.0)	
Developmental Delay	5 (33.3)	1 (2.1)		6 (11.5)	0	
ophthalmologic exam	n = 16	n = 51		n = 57	n = 10	
Abnormal	10 (62.5)	24 (47.1)	0.2811	31 (54.39)	3 (30.0)	0.1548
BERA	n = 16	n = 48		n = 54	n = 10	
Abnormal	3 (18.8)	5 (10.4)	0.3827	6 (11.11)	2 (20.0)	0.4350
Audiometry	n = 15	n = 47		n = 50	n = 12	
Abnormal	1 (6.7)	2 (4.3)	0.7047	3 (6.0)	0	0.3844

SDQ, strengths and difficulties questionnaire.

a Rates of early neurodevelopment (Bayley-III), ophthalmologic abnormalities, and auditory findings in children exposed to Zika with and without abnormal academic and SDQ findings. Analysis used to explore early associations of later challenges.
