# Supplementary material for: Academic and social-behavioral assessment in a prospective cohort of normocephalic school-aged children with antenatal Zika virus exposure
Source: Int J Infect Dis. Author manuscript; Available in PMC 2025 Sep 23. (PMC12453135; doi:10.1016/j.ijid.2025.108026)

**Supplemental Figure 1. Participant Recruitment.** Flowchart illustrating recruitment and grouping of children with antenatal ZIKV exposure and age-matched controls.


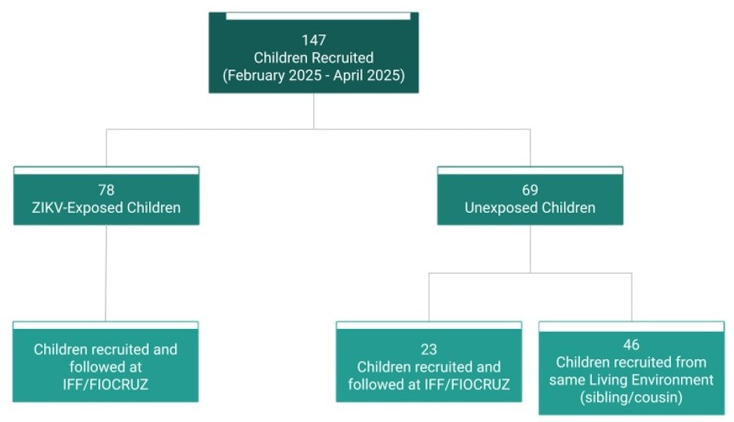

Supplement: Supplemental Figure 1 [file NIHMS2110168-supplement-Supplemental_Figure_1.docx]
