# Supplementary material for: Academic and social-behavioral assessment in a prospective cohort of normocephalic school-aged children with antenatal Zika virus exposure
Source: Int J Infect Dis. Author manuscript; Available in PMC 2025 Sep 23. (PMC12453135; doi:10.1016/j.ijid.2025.108026)
Supplement: Supplemental Material 1 [file NIHMS2110168-supplement-Supplemental_Material_1.docx]

**Supplemental Material 1. Academic Performance Assessment Questionnaire**

**Purpose:** To evaluate children’s academic performance and factors influencing school outcomes. Screening for visual and hearing difficulties is included, as impairments in these areas can affect academic skills and survey responses.

**1. School Information**

- School type: ☐ Public ☐ Private
- Current grade level: _______

**2. Academic Abilities**
*For each domain, indicate if the child is unable to perform expected skills for their grade level based on school evaluations, comparison with peers, and need for additional assistance.*

- Reading difficulties: ☐ Yes ☐ No
- Writing difficulties: ☐ Yes ☐ No
- Mathematical difficulties: ☐ Yes ☐ No

**3. School Attendance**

- Attendance in the past year: ☐ ≥80% ☐ <80%

**4. Neurodevelopmental / Behavioral Evaluations**
*Indicate whether the child has ever undergone evaluation for developmental or behavioral conditions (e.g., intellectual disability, autism spectrum disorder, ADHD, oppositional defiant disorder).*

- Evaluation completed: ☐ Yes ☐ No
- If yes, outcome: ☐ None ☐ Suspected ☐ Diagnosed

**5. Sensory Screening**
*Indicate whether the child has ever undergone hearing or vision assessment. Specify the type of evaluation and the outcome.*

- **Hearing evaluation completed:** ☐ Yes ☐ No
  - Type of evaluation: _________________________
  - Result: ☐ Normal ☐ Problem identified → Specify: _________________________
- **Vision evaluation completed:** ☐ Yes ☐ No
  - Type of evaluation: _________________________
  - Result: ☐ Normal ☐ Problem identified → Specify: _________________________

**Scoring/Interpretation:**

- Academic difficulties are considered present if the child is unable to meet expected skills for grade level.
- Attendance is classified as higher or lower than 80% over the prior year.
- Neurodevelopmental difficulties are classified as “None,” “Suspected,” or “Diagnosed” based on evaluations.
- Visual or hearing impairments are recorded to contextualize academic performance.
