# Supplementary material for: Academic and social-behavioral assessment in a prospective cohort of normocephalic school-aged children with antenatal Zika virus exposure
Source: Int J Infect Dis. Author manuscript; Available in PMC 2025 Sep 23. (PMC12453135; doi:10.1016/j.ijid.2025.108026)
Supplement: Supplemental Table 1 [file NIHMS2110168-supplement-Supplemental_Table_1.docx]

**Supplemental Table 1. Participant Demographics.** Descriptive analysis for ZIKV group and control group.

|  | **Zika Group** n (%) | **Control Group** n (%) |
| --- | --- | --- |
| Number of Children (n=147) | 78 | 69 |
| Age in Years (n=147) |  |  |
| Mean Age | 8.1 | 7.7 |
| Range Age | 7-9 | 5-13 |
| Sex Assigned at Birth (n=147) |  |  |
| Male | 39 (50.0) | 31 (44.9) |
| Gestation (n=144) |  |  |
| Preterm | 14 (18.7) | 9 (13.0) |
| Delivery (n=144) |  |  |
| Vaginal | 21 (28.0) | 30 (43.5) |
| C-Section | 54 (72.0) | 39 (56.5) |
| Maternal Age in Years (n=145) |  |  |
| >35 | 22 (28.2) | 19 (28.4) |
| Maternal Comorbidities (n=147) |  |  |
| Yes | 16 (20.5) | 18 (26.1) |
| Trimester ZIKV Infection (n=78) |  |  |
| Unknown/Asx | 4 (5.1) | - |
| 1st | 22 (28.2) | - |
| 2nd | 38 (48.7) | - |
| 3rd | 14 (18.0) | - |
