# Supplementary material for: Academic and social-behavioral assessment in a prospective cohort of normocephalic school-aged children with antenatal Zika virus exposure
Source: Int J Infect Dis. Author manuscript; Available in PMC 2025 Sep 23. (PMC12453135; doi:10.1016/j.ijid.2025.108026)
Supplement: Supplemental Table 2 [file NIHMS2110168-supplement-Supplemental_Table_2.docx]

**Supplemental Table 2. Strengths and Difficulties Questionnaire (SDQ) Scores Interpretation and Results.** Comparison of SDQ outcomes between ZIKV-exposed and control children.

|  | **Normal** | **Borderline** | **Abnormal** | **ZIKV**  **(mean score)** | **Control**  **(mean score)** | **p-value** |
| --- | --- | --- | --- | --- | --- | --- |
| **Total Difficulties** | 0-13 | 14-16 | 17-40 | 13.32 | 10.10 | **0.0099** |
| **Emotional** | 0-3 | 4 | 5-10 | 4.33 | 2.90 | **0.0011** |
| **Conduct** | 0-2 | 3 | 4-10 | 2.64 | 2.59 | 0.9105 |
| **Hyperactivity** | 0-5 | 6 | 7-10 | 4.95 | 3.41 | **0.0037** |
| **Peer Difficulties** | 0-2 | 3 | 4-10 | 1.40 | 1.20 | 0.5312 |
| **Prosocial Behavior** | 6-10 | 5 | 0-4 | 8.68 | 8.83 | 0.6211 |
